# Malaria and Diabetes

**Published:** 1882-08

**Authors:** 


### Malaria and Diabetes.

At the meeting of the *Academie de Medecine* on November 29th, M. Verneuil communicated six observations that he had made within the year, relative to surgical affections in persons affected simultaneously with glycosuria and malaria, and he presented an extended historical review of the authors who had previously noted this association of diseases. He cited in particular the memoir of Dr. Burdel, presented to the *Academie des Sciences* in 1859, of which the following were the conclusions: 1. There exists a true diabetes in malaria. 2. This diabetes is only ephemeral, that is to say, it appears with the fever, lasts during its stay, and disappears with it. 3. The glycosuria of malarial fever reveals the disturbance which destroys the equilibrium between the cerebro-spinal and sympathetic nervous systems. 4. This explanation, given by Claude Bernard, is confirmed by the clinical facts. 5. The more violent the cases, and intense the fever, the greater the quantity of sugar in the urine. 6. When, on the contrary, the attacks become more numerous and less severe, or, in other words, when the malarial cachexia is established, then the amount of sugar excreted becomes less.

In his notes, M. Burdel announced that he found sugar in the urine in considerable quantity in eighty instances among eighty-six cases of malarial fever; in thirty cases where the fever had become remittent, the sugar was in less quantity, and that in pregnant or nursing women, the sugar is present in large amounts.

M. Verneuil reports the notes of six cases, which lead him to formulate the following conclusions:—

1. Malaria frequently causes glycosuria.

2. This may occur in two forms: the one cotemporary with the febrile attack, and, like it, transient; the other occurring after and independent of the febrile attack, and, unlike the first, of which it is a consequence, is permanent.

3. Permanent glycosuria is especially apt to occur in malarial subjects of arthritic diathesis.

4. Malarial glycosuria appears to be one of the benign forms of diabetes.

5. Intermittent affections occurring in such subjects may take on certain characters of malaria or diabetes, or of both affections at the same time. Traumatic lesions may irritate or aggravate both diatheses, especially that of telluric origin.—*Gaz. Med. de Paris*, Dec. 3, 1881.

The above subject caused an interesting discussion, M. Collin particularly objecting to M. Verneuil's deductions. In the *Journal de Medecine de Paris*, Dec. 17, 1881, the question is subjected to a critical review, of which the following are the conclusions:—

1. The pathogenic influence of malaria can be admitted in a certain number of cases, even though mere coincidence might also be urged; it is probable, however, that this causative influence is not often exerted.

2. In the cases where diabetes is associated with malaria, it should first be proved that some more efficacious etiological condition is not concurrent with the latter.

3. Although the present state of sciences will not permit the affirmation of the frequent occurrence of diabetes of malarial origin, the facts observed by Prof. Verneuil are of sufficient importance to cause attention to be directed anew to this interesting subject.

